# Genomic Surveillance Reveals the Rapid Expansion of the XBB Lineage among Circulating SARS-CoV-2 Omicron Lineages in Southeastern Wisconsin, USA

**DOI:** 10.3390/v15091940

**Published:** 2023-09-16

**Authors:** Arunachalam Ramaiah, Manjeet Khubbar, Katherine Akinyemi, Amy Bauer, Francisco Carranza, Joshua Weiner, Sanjib Bhattacharyya, David Payne, Nandhakumar Balakrishnan

**Affiliations:** 1 City of Milwaukee Health Department, Milwaukee, WI 53202, USA; 2 Georgia Public Health Laboratory, Decatur, GA 30033, USA

**Keywords:** SARS-CoV-2, COVID-19, Omicron, whole-genome sequencing, XBB.1.5, genomic surveillance

## Abstract

SARS-CoV-2 caused a life-threatening COVID-19 pandemic outbreak worldwide. The Southeastern Region of Wisconsin, USA (SERW) includes large urban Milwaukee and six suburban counties, namely Kenosha, Ozaukee, Racine, Walworth, Washington and Waukesha. Due to the lack of detailed SARS-CoV-2 genomic surveillance in the suburban populations of the SERW, whole-genome sequencing was employed to investigate circulating SARS-CoV-2 lineages and characterize dominant XBB lineages among this SERW population from November 2021 to April 2023. For an unbiased data analysis, we combined our 6709 SARS-CoV-2 sequences with 1520 sequences from the same geographical region submitted by other laboratories. Our study shows that SARS-CoV-2 genomes were distributed into 357 lineages/sublineages belonging to 13 clades, of which 88.8% were from Omicron. We document dominant sublineages XBB.1.5 and surging XBB.1.16 and XBB.1.9.1 with a few additional functional mutations in Spike, which are known to contribute to higher viral reproduction, enhanced transmission and immune evasion. Mutational profile assessment of XBB.1.5 Spike identifies 38 defining mutations with high prevalence occurring in 49.8–99.6% of the sequences studied, of which 32 mutations were in three functional domains. Phylogenetic and genetic relatedness between XBB.1.5 sequences reveal potential virus transmission occurring within households and within and between Southeastern Wisconsin counties. A comprehensive phylogeny of XBB.1.5 with global sub-dataset sequences confirms the wide spread of genetically similar SARS-CoV-2 strains within the same geographical area. Altogether, this study identified proportions of circulating Omicron variants and genetic characterization of XBB.1.5 in the SERW population, which helped state and national public health agencies to make compelling mitigation efforts to reduce COVID-19 transmission in the communities and monitor emerging lineages for their impact on diagnostics, treatments and vaccines.

## 1. Introduction

Severe Acute Respiratory Syndrome Coronavirus 2 (SARS-CoV-2) is a positive-sense, single-stranded RNA virus that causes a life-threatening outbreak of Coronavirus disease 2019 (COVID-19) worldwide [1]. A Public Health Emergency of International Concern (PHEIC) for COVID-19 was declared by the World Health Organization (WHO, accessed on 31 May 2023, https://www.who.int/news/item/) from 30 January 2020 through 5 May 2023. New lineages of SARS-CoV-2 continue to evolve through genetic mutations, recombination, immune evasion or viral adaptation to the hosts [2,3], which is an ongoing public health concern. Consistent genetic changes have a profound impact on the physical and biological properties of viruses, leading to emerging variants, including variants of concern (VOC) [4,5,6,7]. As of today, five VOCs, including Alpha, Beta, Gamma, Delta and Omicron, have been identified [8,9], of which the Omicron variant became more prevalent and overtook the other four VOCs.

Since December 2020, the United States Centers for Disease Control and Prevention (CDC) has employed national genomic surveillance to track SARS-CoV-2 lineages at the national level and ten regional levels of the U.S. Department of Health and Human Services (HHS) [10,11,12,13,14]. Each of the ten HHS regions directly serves a range of 4–10 states/islands and local organizations within their jurisdictions. While these reports delineate circulating SARS-CoV-2 lineages broadly, genomic surveillance reports within HHS, specifically at the individual states, regions within states or local populations, would benefit tracking the regional prevalence, emerging VOCs and guiding public health action within their jurisdictions [3,15,16,17,18,19,20]. HHS Region 5 consists of six states, including Wisconsin. The Southeastern Region of Wisconsin, USA (SERW) includes large urban Milwaukee and six suburban counties namely Kenosha, Ozaukee, Racine, Walworth, Washington and Waukesha (Index of Relative Rurality; https://worh.org/, accessed on 24 August 2023). Over the three years of the ongoing COVID-19 pandemic, sustained whole-genome sequencing (WGS) has enabled the City of Milwaukee Health Department Laboratory (MHDL) and other laboratories to identify the circulating SARS-CoV-2 variants, transmission patterns, vaccine breakthrough and contact tracing investigations substantially in a diverse population in Milwaukee [3,15,16,21,22]. However, the lack of detailed SARS-CoV-2 genomic surveillance in those six suburban populations increases the risks of implementing effective public health actionable interventions in SERW during pandemics. For effective disease interventions and informing public health policy in SERW including six suburb populations, it is important to identify circulating and emerging SARS-CoV-2 lineages and the transmission dynamics of dominantly circulating Omicron lineages (i.e., XBB.1.5). Therefore, MHDL has tested a total of 16,179 nasopharyngeal/nasal swab specimens for SARS-CoV-2 collected from SERW between November 2021 and April 2023. Of 16,179 SARS-CoV-2-positive specimens, 50.2% were sequenced for baseline genomic surveillance. Only 82.7% of the generated SARS-CoV-2 sequences that passed post-run quality control (QC) metrics were used for further bioinformatic data analysis. Overall, our efforts for genomic surveillance of SARS-CoV-2 in SERW have identified circulating Omicron lineages, mutational profile, and the transmission patterns of rapidly expanded XBB.1.5 in XBB lineages. This study emphasizes the importance of continuous SARS-CoV-2 genomic surveillance for making appropriate, compelling mitigation efforts to reduce COVID-19 transmission in the communities.

## 2. Materials and Methods

### 2.1. SARS-CoV-2-Positive Clinical Specimens

According to CDC Interim Guidelines for Collecting and Handling of Clinical Specimens for COVID-19 Testing [23], the MHDL received 16,179 nasopharyngeal/nasal swab specimens for SARS-CoV-2 testing from Southeastern Wisconsin between November 2021 and April 2023. Nucleic acid from SARS-CoV-2-positive specimens was extracted using FDA EUA-approved automated extraction platforms [24,25], as per manufacturer’s instructions. The extracts were stored at −80 °C until testing. Of 16,179 SARS-CoV-2-positive nasopharyngeal/nasal swab specimens, 50.2% (*n* = 8116) were sequenced for baseline genomic surveillance [3,15]. Only 82.7% (*n* = 6709/8116) of the generated SARS-CoV-2 sequences (those that passed post-run QC metrics of 100× read depth and 90% genomic coverage) were used for further bioinformatic data analysis as described [3,15]. These 6709 specimens were sequenced using Illumina’s MiSeq, Oxford Nanopore Technologies (ONT)-based Clear Dx or MinION platforms (Table S1). The detailed methods from specimen collection to SARS-CoV-2 data submission to GISAID were provided elsewhere [3,15]. However, here we explain in brief about all three sequencing platforms.

### 2.2. Clear Dx Sequencing Platform

The Clear Dx (Clear Labs, CA, USA) is a fully automated whole-genome sequencing (WGS) platform for SARS-CoV-2 detection and genome sequencing. The workflow begins from extracted RNA to automated library preparation, sequencing and bioinformatic data analysis with minimal human intervention. The reagent preparation, assay processing, and analysis were performed according to the Clear Dx WGS SARS-CoV-2 assay manufacturer’s instructions and MHDL standard operating procedures. The system uses a Hamilton STAR robotic platform for automation of liquid handling and includes required ancillary equipment, such as Hamilton thermal cyclers, a barcode reader, magnet block and two MinION nanopore sequencers from ONT. Nucleic acids extracted from SARS-CoV-2-positive specimens were amplified using ARTIC V3 (biopipeline BIP-Wv6) or MIDNIGHT (biopipeline BIP-WV7) primer pools. Barcode classification of sequencing reads and assembly of the consensus SARS-CoV-2 genome were automated using a modified version of the ARTIC bioinformatics pipeline (BIP-WV6–BIP-WV14) in the Clear Labs WGS App (https://wgs.app.clearlabs.com/) or Medaka via ARTIC 1.2.1 or 1.3.0 in Terra platform (https://terra.bio/). 

### 2.3. MinION Sequencing Platform

The extracted nucleic acids from the clinical specimens were converted into cDNA, and then a PCR tiling protocol was performed to amplify overlapping ‘tiled’ sections covering the SARS-CoV-2 genome using V3 primer pools. It yielded tiled 400 bp amplicons as per ARTIC multiplex PCR protocol [26]. After cDNA amplification, the amplicons were barcoded for sequencing with the Native Barcoding Expansion Pack to prepare the samples for sequencing in a single run and then pooled. After library preparation, the specimens were sequenced in multiplex on MinION flow cells using either a MinION Mk1B or MinION Mk1C device. MinKNOW software v4.1.22 was used for base calling. Demultiplexing of reads was carried out using the EPI2ME Labs ARTIC SARS-CoV-2 workflow (https://github.com/epi2me-labs/wf-artic/). The FASTQ files were processed using the ARTIC bioinformatics pipeline (https://github.com/artic-network/artic-ncov2019) [26] by mapping demultiplexed raw FASTQ files to the reference virus genome Wuhan-Hu-1 using minimap2 [27]. The consensus sequences were generated, and variants were called using Medaka.

### 2.4. MiSeq Sequencing Platform

The Illumina DNA Prep library kit was used on MiSeq platform [28] for sequencing SARS-CoV-2 genome by following the sample preparation procedures used for MinION from converting RNA into cDNA, PCR tiling and primer selection to amplification of targeted regions. Then, sequencing libraries were prepared by adding specialized adapters to both ends of amplicons by tagmentation chemistry. These adapters contain complementary sequences that allow the DNA fragments to bind to the flow cell. Then, fragments were amplified and purified. The multiplex libraries prepared were pooled together and sequenced. The FASTQ files generated from the MiSeq platform were assembled using Illumina DRAGEN COVID Lineage (v3.5.4–v3.5.13), where the reads aligned to a reference genome for calling variants and producing a consensus genome sequence for each specimen. The consensus sequences generated from all three sequencing platforms and related metadata were submitted to GISAID (https://www.gisaid.org/) [29].

### 2.5. Nextclade Assignment and Phylogenetic Analysis of Sequences

The Nextstrain clade and Pango lineage assignment for 6709 consensus SARS-CoV-2 genomes were uniformly performed for this study using Nextclade version web-2.8.1 [30]. We have used Nextclade’s aligned 267 SARS-CoV-2 XBB.1.5 genome sequences under GTR + F + R2 to construct the maximum likelihood tree with 1000 bootstrap replicates in IQ-TREE multi-core version 2.0.3 [31]. The phylogenetic tree was visualized in Interactive Tree Of Life (iTOL) [32]. Prism GraphPad v9.4.1 (www.graphpad.com) was used for generating figures. Audacity*Instant* (v.5.0.1) search was performed against 15.8 million SARS-CoV-2 sequences in GISAID for randomly selected XBB.1.5 strains to identify the closest and related genomes. To identify the phylogenetic relatedness and nature of clustering of XBB.1.5 strains, genome sequences from our dataset and the existing global sub-dataset (Nextstrain, accessed in 2023) were used.

## 3. Results and Discussion

### 3.1. SARS-CoV-2-Positive Clinical Specimens and Associated Metadata

**Figure 1:**
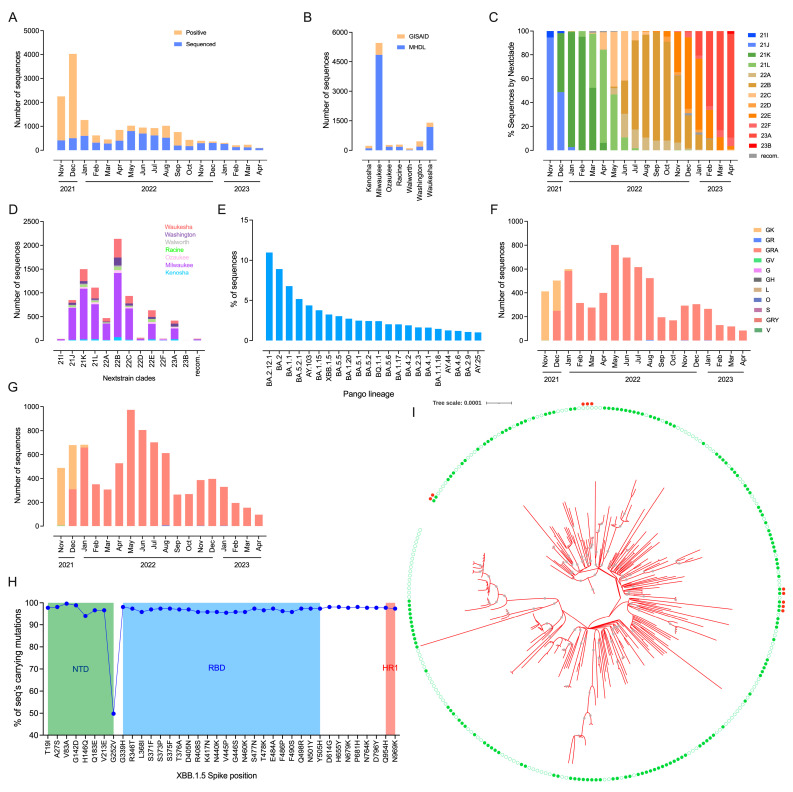
Chronological distributions of SARS-CoV-2 sequences from Southeastern Wisconsin, USA, collected during November 2021–April 2023. (**A**) Total number of SARS-CoV-2-positive samples and the number of samples sequenced (QC passed) from patients on a monthly basis. (**B**) Total number of sequences from each of seven counties in Southeastern Wisconsin. (**C**) Chronological distribution of SARS-CoV-2 genomic variants in the Southeast Wisconsin population. The clades for 8229 genome sequences of SARS-CoV-2 were classified based on the sampling date. Clades are color-coded following the naming convention and branching colors in Nextstrain. (**D**) Classification of a total number of sequences from seven counties based on 13 Nextstrain clades. (**E**) Distribution of 8229 SARS-CoV-2 genomic sequences into 357 Pango lineages/sublineages. Only the lineages/sublineages identified in >1% of the sequences studied are displayed (refer to Table S3 for a full list). Comparison of chronological distribution of SARS-CoV-2 genomic variants sampled from (**F**) Southeastern Wisconsin’s MHDL data and (**G**) Southeastern Wisconsin’s combined MHDL and public data during November 2021–April 2023. (**H**) The mutational profile of 267 Spike sequences of dominant XBB.1.5 lineage. We document a total of 38 high-prevalence defining mutations. Of these, mutations exclusively detected in the *N*-terminal domain (NTD, green), receptor-binding domain (RBD, blue) and heptad repeats-1 domain (HR1, red) were highlighted. (**I**) The maximum likelihood phylogenetic representation of all XBB.1.5 (*n* = 267) SARS-CoV-2 genomes sequenced from clinical specimens of the population in Southeastern Wisconsin. The bootstrap values were displayed in the branches if they were ≥60% supported (grey circles in the middle of the branches). Most of the nodes in the tree were formed with 100% bootstrap supports (large circles). The branch distance corresponds to substitution per site. The metadata are visualized as circles exterior to the branches in the tree. In circle 1 (inner), the green-filled and outlined circle symbols exemplify that the clinical samples used for sequencing originated from Milwaukee County and six other suburban counties in Southeast Wisconsin Region, respectively. In circle 2 (red), the filled circle symbols show the closest relationship of the representative strains possibly responsible for household transmission from Milwaukee and Washington Counties (Please refer to Table S6 for more details).

Of 16,179 SARS-CoV-2-positive specimens, 50.2% (*n* = 8116) were sequenced; however, only 82.7% (*n* = 6709/8116) of the generated SARS-CoV-2 sequences (those that passed post-run quality control (QC) metrics of 100× read depth and 90% genomic coverage) were further analyzed (Figure 1A; Table S1). Here, most of the specimens studied were collected from Milwaukee County (72.2%), followed by Waukesha (17.7%) and Washington (2.8%) Counties (Figure 1B; Table S1). Of 6709 specimens, 59% (3957), 40.9% (2745) and 0.1% (7) were sequenced using MiSeq, Clear Dx and MinION platforms, respectively (Table S1). Metadata show that 8.8% (*n* = 588) of 6709 specimens have no gender listed on the specimen submission form. The majority of specimens (50.3%, 3377) were collected from females in the age group ranging from 1 to 103 years (median age of 38 years) (Table 1 and Table S1). This agrees with the metadata of more than 1.3 million confirmed COVID-19 cases in the United States [33] and 12 million COVID-19 cases recently analyzed worldwide [2], indicating a propensity for females to be susceptible to COVID-19 infection. However, reports show that males experience higher COVID-19 severity and fatality, likely due to higher levels of expression of the Angiotensin-Converting Enzyme 2 (ACE2) receptor and the transmembrane protease serine 2 (TMPRSS2) in males, hormonal influences on the immunological response and behaviors [33,34,35,36,37,38,39,40,41].

### 3.2. Identification of Circulating SARS-CoV-2 Lineages Reveals Rapid Spread of the Virus in Most Densely Populated Milwaukee County

For further descriptive analysis of SERW sequencing data in an unbiased approach, 1520 SARS-CoV-2 sequences from the same geographical region submitted by other clinical laboratories to the GISAID database (GISAID EpiCov v2.5.1; accessed on 30 May 2023) were combined with our MHDL sequencing data (*n* = 6709) (Table S2). Remarkably, MHDL has sequenced 81.5% of specimens submitted to GISAID (*n* = 6709/8229) from SERW. This comprehensive MHDL data set and other clinical laboratories reflect concordance with specimen distribution patterns in most counties (Figure 1B). The clade and lineage assignment (Nextclade v2.14.1; accessed on 1 June 2023) for this data set (*n* = 8229) showed that the genome sequences were distributed into 357 Pango lineages/sublineages belonging to 13 Nextstrain clades (Figure 1C–E; Tables S2 and S3). Of 13 clades, 88.8% (*n* = 7307) of the sequences were derived from ten clades of Omicron, 10.7% (*n* = 883) from two clades of Delta and 0.5% (*n* = 39) belonged to seven recombinant types. Among ten Omicron clades, 22B (BA.5/descendants), 21K (BA.1/descendants) and 21L (BA.2/descendants) were most commonly identified in >57% of the specimens studied. Of the seven recombinant types identified, XAE and XBK types were detected from March to April 2022 and November 2022 to February 2023, respectively, and most recently, XBF in April 2023. 

During the study period, SARS-CoV-2 strains belonging to all 13 clades were found to be circulating in Milwaukee and Waukesha Counties; however, strains belonging to a total of 1–5 clades (21I, 22D, 22F, 23B, recombinant) have not been detected in the specimens from other five suburban counties (Figure 1D). The two-tailed t-testing of the prevalence of these 13 clades in the large Milwaukee County and six other suburban counties showed significant differences (*p*-value < 0.01). Similarly, Chi-square testing of these 13 clades in SERW exhibited significant differences (*p*-value <0.0001). We observed that the Delta variant decreased (47.7%, 883) from November 2021 to January 2022, with the subsequent emergence of the Omicron variant (21K, BA.1.17) in December 2021 (Figure 1C). Seven (21K, Omicron) lineage/sublineages, including BA.1.17, BA.1.15, BA.1.20, BA.1, BA.1.1.18, BA.1.1 and BA.1.1.14, were first detected in December 2021. Interestingly, all these specimens were collected specifically from patients in Milwaukee County, indicating the rapid spread of the virus is within Wisconsin’s most densely populated county. Further investigation of currently circulating lineages shows that BQ.1 (22E/Omicron) was detected on 30 September 2022 in Milwaukee, and subsequently, descendant lineages were detected and continue to circulate in SERW. However, BQ.1 was first detected in Wisconsin state on 21 September 2022, which is more than a week prior to the date of detection in SERW, indicating BQ.1 appears to be introduced first in another region of Wisconsin. Within the 22E clade, BQ.1.1 (31.3%) and BQ.1.1.18 (10.2%) were the most established sublineages detected first in October 2022 in Racine and Washington Counties, respectively. Analysis of XBB clades (22F, 23A, 23B) shows that new lineages appeared to have been first introduced in Milwaukee on 23 November 2022 (i.e., XBB.1, XBB.1.9.1, XBB.1.15, XBB.1.5, XBB.1.5.1, XBB.1.5.15, XBB.1.5.19, XBB.1.5.52) and have appeared in other counties since 16 December 2022 (i.e., XBB.1.16, XBB.1.5.11, XBB.1.5.13, XBB.1.5.16, XBB.1.5.2). Since February 2022, the Omicron variant has accounted for 100% of the circulating variants in SERW. We observed that this pattern of Omicron variant evolution in SERW was consistent with the Wisconsin state and national trends (Figure 1F,G and Figure S1; Table S4). 

### 3.3. Mutational Profile and Transmission Dynamics of Rapidly Expanded XBB.1.5 Lineage in Southeastern Wisconsin Population

As for the rapidly expanded XBB.1.5 lineage being the leading cause of morbidity in the current phase of COVID-19, we studied it further along with surging lineages in the community. Our findings documented dominant XBB.1.5 (23A, Omicron), surging XBB.1.16 (23B) and XBB.1.9.1 (22F) sublineages in 58%, 0.2% and 0.4% of the specimens among XBB clades with a range of 69–107, 104 and 99–101 mutations, respectively. This indicates the evolutionary trajectory of the XBB strains through consistent mutations over the course of the pandemic that can potentially change with the viral pathogenesis. Remarkably, these sequences exhibited a few additional functional mutations in Spike (i.e., XBB.1.5-G252V, F486P; XBB.1.16-F486P) and other proteins (i.e., XBB.1.5-ORF8: G8*; XBB.1.16-ORF8: G8*, -ORF1a: L3829F, -ORF1b: D1746Y, -ORF9b: I5T; XBB.1.9.1-ORF8: G8*), which are known to be contributing for the higher reproductive rate, enhanced transmission, immune evasion, lowering binding affinity of receptor-binding domain (RBD) to ACE2 and resistant to monoclonal and neutralizing antibodies [42,43,44,45,46]. As of 12 June 2023, these three variants account for about 70% of cases in the United States. Considering the vital function of Spike for viral entry into the host cell, we assessed the mutational profile of 267 Spike sequences of dominant XBB.1.5 lineage in our comprehensive data set. The analysis revealed 77 non-synonymous mutations, including 38 defining mutations with high prevalence occurring in 49.8–99.6% of the sequences studied (Figure 1H; Table S5). Among these 77, 18 (8 defining mutations, e.g., T19I, A27S), 34 (22; e.g., R346T, N460K) and 2 (2; Q954H, N969K) mutations were detected in the *N*-terminal domain (NTD), RBD, and heptad repeats-1 domain (HR1), respectively. Metadata and genomic analysis show that except XBB.1.16 (Waukesha, 24 April 2023), the other two lineages, XBB.1.5 and XBB.1.9.1, were detected in Milwaukee on 27 December 2022 and 28 March 2023, respectively. However, these three lineages were first detected in other regions of Wisconsin state 2–4 weeks prior to the date of detection in SERW, indicating the possible interregional transmission of these lineages. This finding necessitates public health agencies to monitor these XBB sublineages and verify the impact of these amino acid substitutions on currently available monoclonal antibody therapeutics.

Using maximum likelihood phylogenetic and genetic sequence comparison approaches, we identified the transmissibility of specific XBB.1.5 strains circulating in the SERW populations. These analyses with associated metadata identified that potential virus transmission might have occurred within a household (i.e., Milwaukee: EPI_ISL_16454816 and EPI_ISL_16543797), as well as within (i.e., Milwaukee: EPI_ISL_16841073 and EPI_ISL_16841060) and between Southeastern Wisconsin counties (i.e., Milwaukee: EPI_ISL_16543845 and Washington: EPI_ISL_16845919) (Figure 1I; Table S6). Further pairwise comparison of these sequences identified a 0–1 mutation difference in all regions with coverage. Our data also reveal that specific strains in SERW (i.e., Milwaukee, EPI_ISL_16841071) prior to the first widespread detection in other regions of Wisconsin, and vice versa (i.e., Washington, EPI_ISL_16611158), elucidating transmission dynamics of specific XBB.1.5 strains. Additional, comprehensive phylogeny based on the SERW and global sub-dataset XBB.1.5 sequences exhibits that the sequences from the SERW populations were closely related (Figure S2), thereby confirming the widespread transmission of genetically similar SARS-CoV-2 strains within the same geographical area [3].

## 4. Conclusions

The SARS-CoV-2 genetic sequences generated by MHDL and other clinical laboratories for baseline molecular surveillance have been efficiently used for identifying proportions of circulating variants and rapid expansion XBB lineages in Southeastern Wisconsin’s urban and suburban populations. The MHDL’s SARS-CoV-2 genomic surveillance data have assisted (i) public health agencies such as the Wisconsin Department of Health Services and the U.S. CDC to make compelling mitigation efforts to reduce COVID-19 transmission in the communities [3] and (ii) closely monitor emerging variants for their impact on diagnostics, treatments and vaccines. One limitation of this study is that the sequences reported in this manuscript represent only a subset of the SARS-CoV-2-positive cases in SERW. This study also emphasizes the importance of continuous SARS-CoV-2 genomic surveillance for appropriate public health mitigation and policy efforts, as this virus is expected to become seasonally endemic.

## Figures and Tables

**Table 1:** Characteristics of 6709 COVID-19 Patients from Southeastern Wisconsin, USA, November 2021–Apil 2023.

Features	MHDL Data
No. of SARS-CoV-2 specimens sequenced (QC passed)	6709
Gender	
Female	3377
Male	2744
Unknown	588
Age *	
Female	1–103 (38)
Male	1–99 (36)
Unknown	1–95 (39)

* Value in parenthesis is median. Age 1–12 months is considered as 1 year.

## Data Availability

All 6709 SARS-CoV-2 genomes sequenced in the MHDL have been deposited with the Global Initiative on Sharing All Influenza Data (GISAID, https://www.gisaid.org/). The accession numbers for all these sequences are provided in Tables S1 and S4.

## Supplementary Materials

The following supporting information can be downloaded at https://www.mdpi.com/article/10.3390/v15091940/s1. Figure S1: Comparison of the chronological distribution of SARS-CoV-2 genomic variants sampled from (A) Wisconsin State’s and (B) the USA’s populations during November 2021–April 2023; Figure S2: Identification of clustering of genetically closely related XBB.1.5 sequences from Southeastern Wisconsin, USA. (A) The phylogenetic analysis of the XBB.1.5 sequences from Southeastern Wisconsin and global sub-data set (Nextstrain, accessed on 24 July 2023; refer to Table S4 for accession numbers and other details). Before constructing the tree, these sequences aligned against the reference strain Wuhan-Hu-1/2019. Expanded form of the representative phylogenetic clusters of (B) Southeastern Wisconsin sequences (highlighted in rectangle), (C) South Africa and (D) Columbia, highlighting the sequences of the same lineages from respective populations were more closely related. Table S1: Details of 6709 SARS-CoV-2 genomes sequenced from nasopharyngeal/nasal swab specimens in the Milwaukee Health Department Laboratory (MHDL), November 2021–April 2023. All these clinical specimens were collected from Southeastern Wisconsin. Refer to the xlsx file. Table S2: Details of 8229 SARS-CoV-2 genome sequences from Southeastern Wisconsin, November 2021–April 2023. Refer to the xlsx file. Table S3: Distribution of 8229 SARS-CoV-2 genomic sequences into 357 lineages/sublineages. Refer to the xlsx file. Table S4: Acknowledgment of Data Contributors. The hyperlinks provided can be used to access the data contributor details and GISAID’s EpiCoV database accession IDs of the SARS-CoV-2 genome sequences from samples of (A) Southeast Wisconsin (6709 specimens sequenced at MHDL), (B) Southeast Wisconsin (8229 sequences include MHDL and GISAID public data), (C) State of Wisconsin, (D) United States populations, collected from November 2021 to April 2023. (E) SARS-CoV-2 sequences subsampled from a global dataset. Refer to the xlsx file. Table S5: The mutational profile of 267 Spike sequences of dominant XBB.1.5 lineage identified in our comprehensive data set. Refer to the xlsx file. Table S6: Details of genetically closest and related genomes of randomly selected 30 XBB.1.5 genomes from the Southeastern Wisconsin population. Refer to the xlsx file.
