# Supplementary material for: Genomic Surveillance Reveals the Rapid Expansion of the XBB Lineage among Circulating SARS-CoV-2 Omicron Lineages in Southeastern Wisconsin, USA
Source: Viruses. 2023 Sep 16;15(9):1940. doi: 10.3390/v15091940 (PMC10535685; doi:10.3390/v15091940)
Supplement: Supplementary file 1 [file viruses-15-01940-s001.zip › Supplemental Figures.pdf]

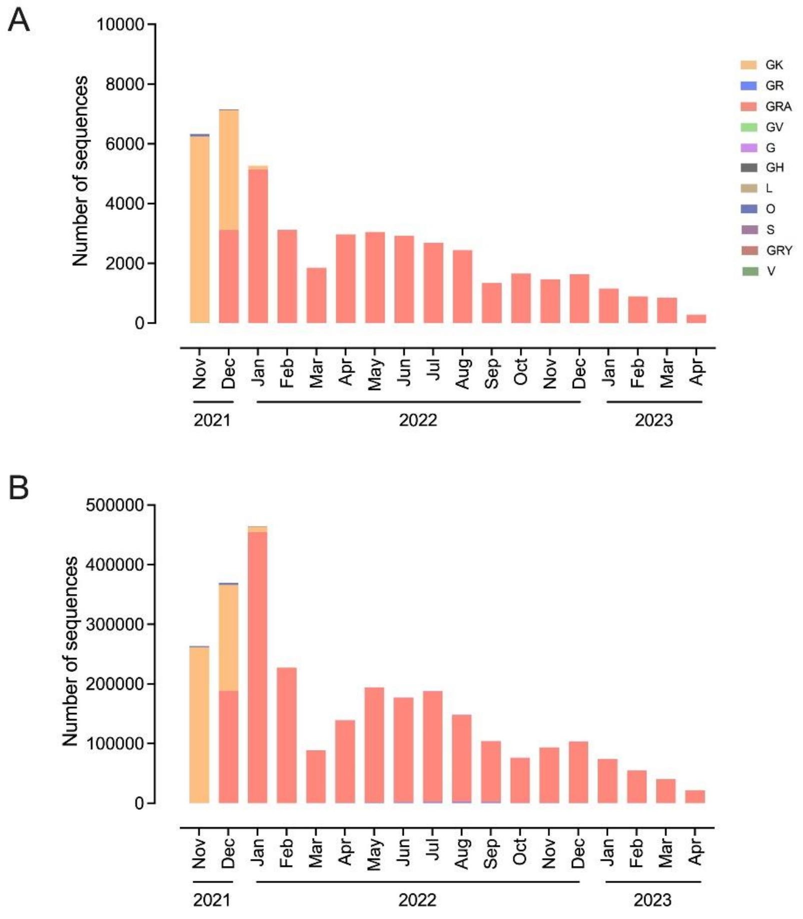

Figure S1. - Comparison of chronological distribution of SARS-CoV-2 genomic variants sampled from, (A) Wisconsin State's, and (B) the USA's populations during November 2021–April 2023.

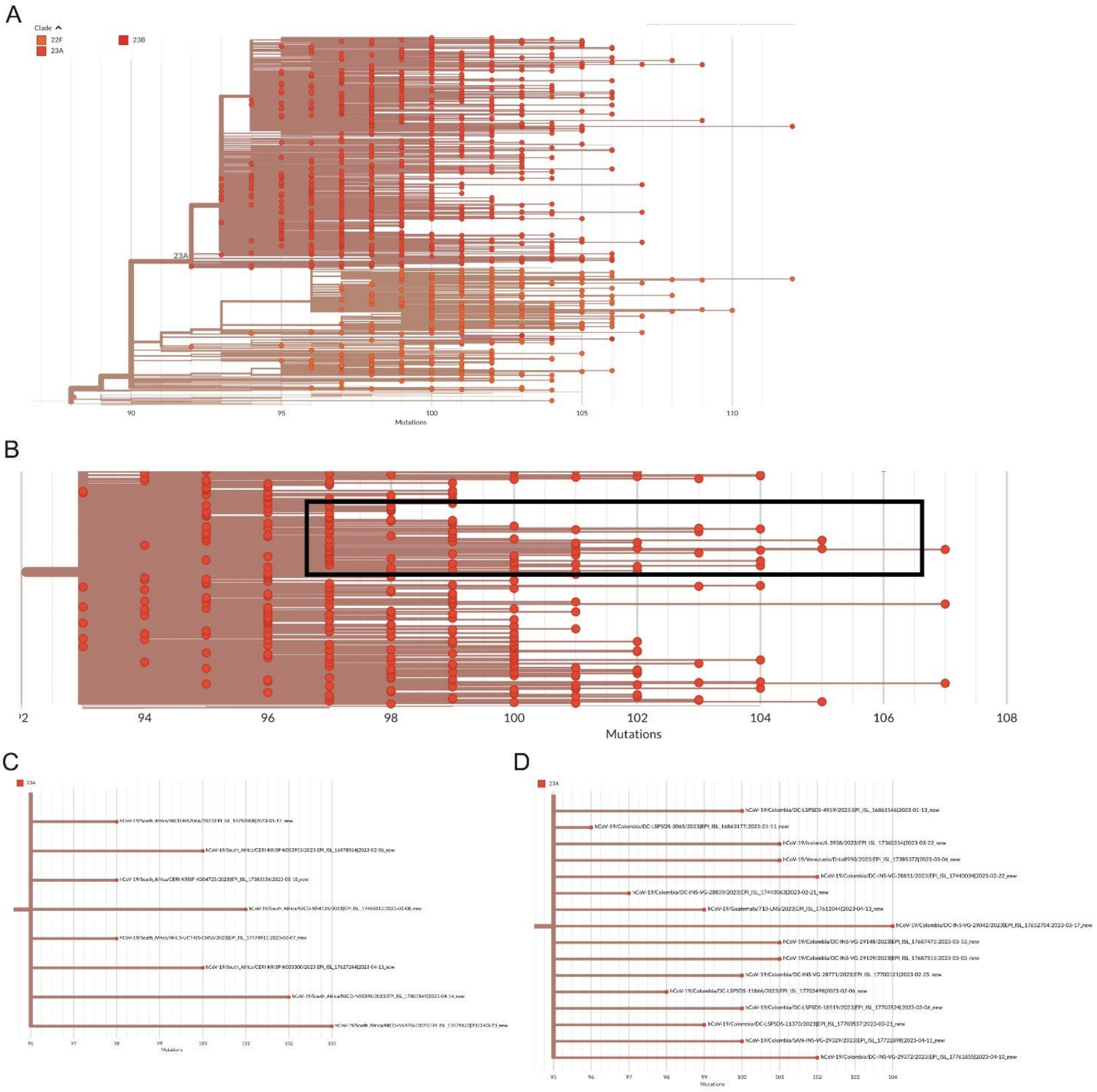

Figure S2. - Identification of clustering of genetically closely related XBB.1.5 sequences from Southeastern Wisconsin, USA. (A) The phylogenetic analysis of the XBB.1.5 sequences from Southeastern Wisconsin and global sub-data set (Nextstrain, accessed on 24 July 2023; refer to Table S4 for accession numbers and other details). Before constructing the tree, these sequences aligned against the reference strain Wuhan-Hu-1/2019. Expanded form of the representative phylogenetic clusters of (B) Southeastern Wisconsin sequences (highlighted in rectangle), (C) South Africa, and (D) Columbia, highlighting the sequences of the same lineages from respective populations, were more closely related.
